# Receptor residence time trumps drug-likeness and oral bioavailability in determining efficacy of complement C5a antagonists

**DOI:** 10.1038/srep24575

**Published:** 2016-04-20

**Authors:** Vernon Seow, Junxian Lim, Adam J. Cotterell, Mei-Kwan Yau, Weijun Xu, Rink-Jan Lohman, W. Mei Kok, Martin J. Stoermer, Matthew J. Sweet, Robert C. Reid, Jacky Y. Suen, David P. Fairlie

**Affiliations:** 1 Division of Chemistry and Structural Biology, Institute for Molecular Bioscience, The University of Queensland, Brisbane, QLD 4072, Australia; 2 Division of Cell Biology and Molecular Medicine, Institute for Molecular Bioscience, The University of Queensland, Brisbane, QLD 4072, Australia; 3 Centre for Inflammation and Disease Research, Institute for Molecular Bioscience, The University of Queensland, Brisbane, QLD 4072, Australia; 4 ARC Centre of Excellence in Advanced Molecular Imaging, Institute for Molecular Bioscience, The University of Queensland, Brisbane, QLD 4072, Australia

## Abstract

Drug discovery and translation are normally based on optimizing efficacy by increasing receptor affinity, functional potency, drug-likeness (rule-of-five compliance) and oral bioavailability. Here we demonstrate that residence time of a compound on its receptor has an overriding influence on efficacy, exemplified for antagonists of inflammatory protein complement C5a that activates immune cells and promotes disease. Three equipotent antagonists (3D53, W54011, JJ47) of inflammatory responses to C5a (3nM) were compared for drug-likeness, receptor affinity and antagonist potency in human macrophages, and anti-inflammatory efficacy in rats. Only the least drug-like antagonist (3D53) maintained potency in cells against higher C5a concentrations and had a much longer duration of action (*t*_1/2_ ~ 20 h) than W54011 or JJ47 (*t*_1/2_ ~ 1–3 h) in inhibiting macrophage responses. The unusually long residence time of 3D53 on its receptor was mechanistically probed by molecular dynamics simulations, which revealed long-lasting interactions that trap the antagonist within the receptor. Despite negligible oral bioavailability, 3D53 was much more orally efficacious than W54011 or JJ47 in preventing repeated agonist insults to induce rat paw oedema over 24 h. Thus, residence time on a receptor can trump drug-likeness in determining efficacy, even oral efficacy, of pharmacological agents.

Drug discovery, development and translation have relied heavily upon optimizing ligand affinity for a specific receptor, enhancing functional potency and selectivity, and improving drug-like properties for optimal pharmacokinetics and oral bioavailability. Over the past two decades, the search for new pharmaceuticals has been heavily constrained by the imposition of rigorous rules, originally proposed only as guidelines based on empirical experimental observations^1^,^2^,^3^, for developing orally bioavailable drugs. For example, compounds with low molecular weight (MW <500), few hydrogen bond donors (HBD ≤5) and acceptors (HBA ≤10), moderate calculated octanol-water partition coefficients (ClogP 1–5), few rotatable bonds (n <12) and low polar surface area (tPSA < 140 Å^2^) are thought to give the best chances of producing orally bioavailable compounds suitable for clinical development^1^,^2^,^3^. On the one hand these ‘rules’ have been extremely valuable and led to many translational successes in the clinic. On the other hand they have become so popular that researchers and companies usually now dismiss out of hand those compounds that violate these rules as being poor choices as drug leads for further development.

Here we debunk the almost universal perception that drugs need to be developed by increasing receptor affinity, potency, drug-likeness (rule-of-five compliance) and oral bioavailability. We focus on a G protein-coupled receptor (GPCR) as the target, this class accounting for around one third of all small molecule pharmaceuticals in the marketplace. We compare three antagonists with equal potency *in vitro* against activation of a GPCR and find that two compounds which are orally bioavailable and strictly obey the drug-likeness guidelines have inferior efficacy, even oral efficacy, to a third compound that comprehensively violates these rules and has dramatically reduced oral bioavailability. We propose that violating rule-of-five and related parameters should not automatically rule out candidates for drug development. Here we show a key factor, the residence time of the ligand on the receptor, that can be even more important for conferring drug efficacy, even via oral administration, and can compensate for perceived deficiencies in drug-likeness and oral bioavailability.

Complement component C5a is a potent proinflammatory and chemotactic factor that primarily signals via the GPCR, C5a receptor 1 (C5aR) on leukocytes. C5aR is expressed widely on immune cells, including neutrophils, monocytes, macrophages, eosinophils and T cells, but also on other cells including of the liver, kidney, adipose, and central nervous system^4^. C5aR signalling is now implicated in many functions besides immunity and inflammation, such as metabolic functions and dysfunction^5^, crosstalk with TLR signalling^6^, developmental biology, and cancer metastasis and progression^7^,^8^. Complement activation is usually tightly regulated during normal physiology, but excessive complement activation can lead to an overproduction of C5a and to inflammatory and autoimmune disorders^9^. Thus, it may be desirable to modulate complement activation using therapeutic interventions such as inhibitors or antibodies. Antibodies that block proteolysis of C5 to C5a and C5b have been FDA-approved for treating paroxysmal nocturnal hemoglobinuria^10^, although blocking C5 (unlike C5a) also prevents downstream formation of the membrane attack complex that promotes lysis and clearance of pathogens and infected or damaged cells thereby compromising immunity. Protein-based inhibitors are also expensive, need to be injected, have poor tissue penetration, and can trigger immunogenic side effects. Unlike proteins and antibodies, drug-like small molecules do not share these disadvantages, but none have yet advanced through clinical trials. To date only a few potent small molecule antagonists of C5aR have been reported^4^,^11^,^12^,^13^,^14^,^15^ with activity in animal models of disease.

Three C5aR antagonists 3D53^4^,^11^,^12^,^13^, W54011^14^ and JJ47^15^ (Fig. 1) are compared here for antagonist potency, antagonist mechanism and duration of action in inhibiting C5aR-mediated human macrophage functions (calcium release, chemotaxis, inflammatory gene expression) and rat paw inflammation. 3D53 is a cyclic peptide designed in our laboratory^4^,^11^,^12^,^13^ on the back of initial peptide studies at Abbott^16^ and Merck^17^. It has been licensed as PMX53 and is safe and well tolerated in Phase I and II clinical trials. All three compounds are orally active, but W54011 and JJ47 are much more drug-like and rule-of-five compliant^1^,^2^,^3^ small organic compounds (Fig. 1). However, despite being less drug-like, comprehensively violating the rule-of-five, and being much less orally bioavailable, the cyclic peptide 3D53 is shown here to be far more efficacious, even when administered orally. This study demonstrates an important lesson in drug discovery and development, that ligand residence time on its receptor can trump rule-of-five considerations and be an overriding feature in dictating drug efficacy *in vitro* and *in vivo,* even oral efficacy for compounds with vastly inferior oral bioavailability. Our study highlights the need for more sophistication now in approaching drug discovery and development in order to successfully translate compounds to market.

## Results

### Comparative antagonism of C5aR

Comparative antagonist potencies and mechanisms under identical conditions were investigated here for the three chemical probes (3D53, W54011, JJ47) in human monocyte-derived macrophages (HMDM). In competitive radioligand-binding experiments using recombinant human ^125^I-C5a, the binding affinities of 3D53 and W54011 for HMDM were comparable, and only slightly weaker for JJ47 (Fig. 2A–C). The concentration-response curves for calcium mobilization induced by rhC5a were determined in the presence of escalating concentrations of each of the three antagonists (Fig. 2D–F). A reduction of the maximal C5a responses was observed as the concentration of 3D53 increased, but there was no rightward shift of the curve typical of competitive or surmountable antagonism, consistent instead with insurmountable C5aR antagonism by 3D53 (Fig. 2D). By contrast, both W54011 and JJ47 were dependent on the C5a concentration. Both caused a rightward shift in concentration-response curves for C5a-induced calcium release in HMDM, without depressing the maximal responses, indicating that both compounds were surmountable antagonists (Fig. 2E,F). A Schild plot and pA_2_ analysis revealed a slope of 0.5 for 3D53 (Fig. 2G), indicating it as a non-competitive rather than competitive antagonist^18^. On the other hand, the slopes for W54011 (Fig. 2H) and JJ47 (Fig. 2I) were both ~1, consistent with these compounds being competitive antagonists with C5a on HMDM and antagonist IC_50_ values being dependent on the concentration of C5a used.

The antagonist IC_50_ value for 3D53 was independent of C5a concentration, maintaining potency against low (1 nM) and high (300 nM) concentrations of C5a (Fig. 3A). On the other hand, W54011 lost most of its antagonist activity against 100 nM C5a (Fig. 3B), with JJ47 being even less potent and losing its antagonist activity against just 10 nM C5a (Fig. 3C). Clearly, W54011 and JJ47 are only antagonists if measured against very low concentrations of C5a, and are unlikely to be very effective *in vivo* under pathophysiological conditions. Accordingly, for inflammatory diseases such as sepsis^19^ or for chronic disease where concentrations of C5a can be as high as 10–100 nM, neither W54011 nor JJ47 would be expected to be very effective antagonists.

### C5a-induced chemotaxis is inhibited most effectively by 3D53

Insurmountable antagonists with a long residence time (slow off-rate) from the receptor-binding site may not reach a true equilibrium between the agonist and antagonist on the receptor in the short timeframe of the calcium release assay (i.e. <5 min). Since C5a is one of the most potent endogenous agents known to induce chemotaxis of immune cells, a more stringent and longer duration test of surmountability is a chemotaxis migration assay, which was performed to investigate the C5aR antagonist mechanism. The antagonist-treated HMDM were exposed to C5a for 16 h continuously. Antagonists with a short residence time will be ineffective in this assay, because once the antagonist has dissociated from C5aR, C5a immediately binds and induces migration. While 3D53 was able to block C5a-induced migration at a concentration of 100 nM, W54011 and JJ47 were not effective antagonists even at 1 μM concentrations (Fig. 4A) over this 16 h timeframe. Since W54011 and JJ47 failed to block chemotaxis here, it seems unlikely that these antagonists would be functional *in vivo* in blocking chemotaxis.

### Extended receptor residence time of 3D53

Residence time refers to the duration that a compound (ligand) is bound to its target and is usually determined by measuring ‘on’ and ‘off’ kinetic rates. Drugs with long residence times potentially offer better selectivity, lower toxicity and a broader therapeutic window^20^. To determine the functional residence time of the three antagonists, washout cell experiments were performed in which HMDM were pre-treated with a saturating concentration of each antagonist (1 μM) for 1 h. HMDM were washed after 1 h to remove unbound antagonist, and then these antagonist-treated HMDM were subsequently challenged with 3 nM C5a at specified time points. From these temporal experiments W54011 and JJ47 were observed to display only short-term antagonist action, with *t*_1/2_ = 1.2 h and 0.6 h, respectively (Fig. 4B). On the other hand, 3D53 maintained a much longer duration of action, *t*_1/2_ = 18.2 h (Fig. 4C). These comparative data highlight the strikingly longer residence time of 3D53 as compared to W54011 and JJ47 on C5aR of HMDM.

### Antagonism of C5a-induced inflammatory gene expression in human and rat macrophages

Real-time PCR analyses were conducted to further check the effect of residence time in other assays. All three antagonists were found to block C5a-induced expression of the inflammatory genes *TNF*, *IL1B, CCL3* and *PTGS2* in HMDM at 1 h post-antagonism, while W54011 and JJ47 blocked C5a-induced *CCL3* gene expression to a lesser extent (Fig. 5A). At 16 h post-antagonist treatment, 3D53 still blocked or significantly inhibited C5a-mediated expression of these human genes, whereas W54011 and JJ47 did not (Fig. 5B). Together, these findings are consistent with W54011 and JJ47 having much shorter durations of action due to much shorter residence times on C5aR of HMDM, being completely displaced from the receptor well within 16 h. Although the responses from human C5a on rat macrophages were not as potent across the same panel of genes, all three antagonists were able to block C5a-mediated responses at 1 h post-antagonism (Fig. 5C). This suggested the feasibility of comparing *in vivo* antagonist activity of 3D53, W54011 and JJ47 in an animal model of inflammation.

### Molecular dynamics simulations of C5aR-3D53 complex

To investigate a possible mechanism accounting for the long residence time of 3D53 on the receptor C5aR, molecular dynamics (MD) simulations were performed for the C5aR-3D53 complex derived from a homology model of C5aR built from an antagonist-CXCR4 crystal structure^21^ (PDB code: 3ODU). After 0–10 ns simulation (Fig. 6A), Arg6 of 3D53 faced extracellular loop 2 (ECL2) of C5aR making a short-lived contact with the Cys188 backbone carbonyl oxygen. After 15 ns, Arg6 moved closer to transmembrane helix TM7 and began to form hydrogen bonds with Asp282 (Fig. 6B). This interaction remarkably closed the distance between the sidechains of these residues from 15 Å to 2 Å (Fig. 6B,D). During intermediate MD simulations, Arg6 of 3D53 sandwiched between TM2 and TM7 (TM2-TM7_dist_ 13 Å to 6.5 Å, Fig. 6D), forming stable hydrogen bonds with the backbone oxygen of Ile91 and the sidechain of Asp282 (Fig. 6C). This led to a stable salt-bridge throughout the later 85 ns and constituted the most stable protein-ligand contact observed (Supplementary Fig. 1). Previous results supported an interaction between Arg6 of 3D53 and Asp282, since Asp282Ala mutation caused a 10-fold loss in affinity of 3D53^22^. In MD simulations, Arg175 and Glu199 also maintained hydrogen bond contact (Fig. 6B,C), suggesting stabilization of an inactive C5aR conformation. Trp5 of 3D53 was found to switch between aromatic pi-interactions and a H-bond interaction with Tyr290, the distance between the NH from the indole ring and the hydroxyl group of Tyr290 being monitored in Fig. 6D.

In early stages of the MD simulations, Trp5 was surrounded by Trp255^6.48^, Tyr258^6.51^ and Tyr290^7.43^ that formed a hydrophobic cage (Supplementary Fig. 2). This observation is consistent with previous structure-activity relationships which found that an aromatic residue (Trp or Phe or Naphthylalanine) at position 5 of 3D53 and its analogues was essential for conferring antagonism and its analogues, whereas non-aromatic residues large or small conferred agonist activity^13^. Indeed MD simulations (20 ns–40 ns) reflected pi-stacking of the indole ring of Trp5 between TM6 (Tyr258) and TM7 (Tyr290) (Supplementary Fig. 3), but after this timeframe Trp5 moved slightly outwards to form a H-bond with Tyr290 in another long-lasting interaction (50 ns–100 ns). Hence, Trp5 appears to play a dual role in conferring both antagonism and a long residence time for 3D53 on C5aR. The corresponding Tyr^7.43^ residue in other GPCRs has also been proposed to be important for activation of GPCRs, including angiostensin-II type 1 receptor and M2 muscarinic receptor^23^,^24^,^25^. Hence, interaction of receptor residue Tyr290^7.43^ with Trp5 of 3D53 may help stabilize C5aR in an inactive conformation. Accordingly, the RMSF plot (Fig. 6E) indicated that the sidechain of the conserved “toggle switch” Trp255^6.48^ of C5aR was lower than 1.0 Å and, together with the conserved NPxxY motif on TM7 (RMSF < 1.0 Å, shown in Fig. 6E), suggested that the C5aR-3D53 complex maintained an inactive conformation throughout the 100 ns simulations.

It is well documented that during GPCR activation, TM3, TM5, TM6 adopt large conformational changes and play important roles in the activation of the receptor^26^,^27^. The lack of such TM conformational change in our MD simulations (Supplementary Fig. 4) suggests that C5aR is locked in an inactive conformation by 3D53. This finding of a long-lasting locked conformation of the 3D53-C5aR complex supports previous mutagenesis results for key residues in C5aR^22^ and provides novel insights into the possible antagonist mechanism accounting for insurmountability and long residence time of 3D53 on C5aR.

### Inhibition of C5aR-PA-induced rat paw oedema

Exogenous recombinant C5a protein is rapidly metabolized in serum within seconds-minutes to C5a-des Arg by carboxypeptidases, which remove the C-terminal arginine that is important for high affinity binding to C5aR^28^. Consequently, C5adesArg has 10–1000 times lower agonist potency than C5a depending upon the function being measured and cell type examined^29^. The very rapid degradation of recombinant C5a protein precludes its use *in vivo* as an agonist. Since only 6–8 residues at the C-terminus of C5a are responsible for agonist activity, although the remainder contribute to high affinity binding, a hexapeptide derivative of the C-terminus (Ac-FKP-dChaCha-dR-OH, referred to as C5aR-PA) was instead used for inducing C5aR-mediated paw oedema in rats. This hexapeptide is a more stable agonist than C5a but with a comparable spectrum of functional C5aR-mediated responses^17^,^30^. This enabled comparison of the specific C5aR antagonists 3D53, W54011 and JJ47 for their effectiveness in inhibiting paw oedema induced by C5aR activation *in vivo* (Fig. 7).

C5aR-mediated rat paw oedema was induced by intraplantar administration of 350 μg C5aR-PA per hind paw (Supplementary Fig. 5A). To inhibit C5aR-PA-induced paw swelling, 3D53 (5 or 10 mg/kg) was given by oral gavage prior to C5aR-PA injection, this reducing paw swelling in a sustained effect at 2 h post-injection of agonist (Supplementary Fig. 5B). On the other hand, W54011 at 5 mg/kg per oral did not block C5aR-PA-induced paw swelling. Even at 10 mg/kg, W54011 did not attenuate paw swelling until 1 h post-agonist injection, and even this effect was quickly lost by 2 h post-injection (Supplementary Fig. 5C). W54011 has been reported to block C5a-induced gerbil neutropenia at concentrations ranging from 3–30 mg/kg^14^. However, W54011 at 10 mg/kg did not have much effect, so the dose was increased to 30 mg/kg. No *in vivo* data for JJ47 was available from the literature, so rats were treated orally at the same dose as for W54011 (30 mg/kg/p.o.). Wistar rats were given 3D53 at 5 mg/kg/p.o., W54011 and JJ47 at 30 mg/kg/p.o., all 1 h prior to intraplantar injection into paws with 350 μg C5aR-PA. Animals were removed from each group at the indicated times and challenged with the localized injection of C5aR-PA into a paw. Even though 3D53 was only given at 5 mg/kg/p.o., compared to W54011 and JJ47 at 30 mg/kg/p.o., its systemic delivery was able to inhibit paw swelling from 2–16 h (Fig. 7A). At 30 min post-injection, W54011 was more effective than 3D53 and JJ47 with 35% reduction in paw swelling (Fig. 7B). However, W54011 was no longer effective after 2 h. Interestingly, JJ47 only caused 15% reduction in paw swelling at 30 min (Fig. 7C) and was not much different to W54011 after 2 h. Clearly, 3D53 was a far superior inhibitor of C5aR-mediated paw inflammation than the other two compounds, with strong anti-inflammatory action still maintained at 16 h post-administration by oral gavage.

Comparative oral bioavailabilities (Fig. 7D–F and Supplementary Table 1) determined in male Wistar rats and measured under identical conditions gave the rank order W54011 (F ~ 74%) > JJ47 (F ~ 12%) > 3D53 (F ~ 2%), the same trend also being observed for maximal plasma concentration (C_max_) and plasma half-life (*t*_1/2_). This finding highlights the remarkable oral efficacy of 3D53 in spite of much lower oral bioavailability than the other two antagonists, its much longer receptor residence time overcoming substantial perceived liabilities associated with its inferior drug-likeness and making it more orally active and a superior antagonist of C5aR.

## Discussion

**Figure 1:**
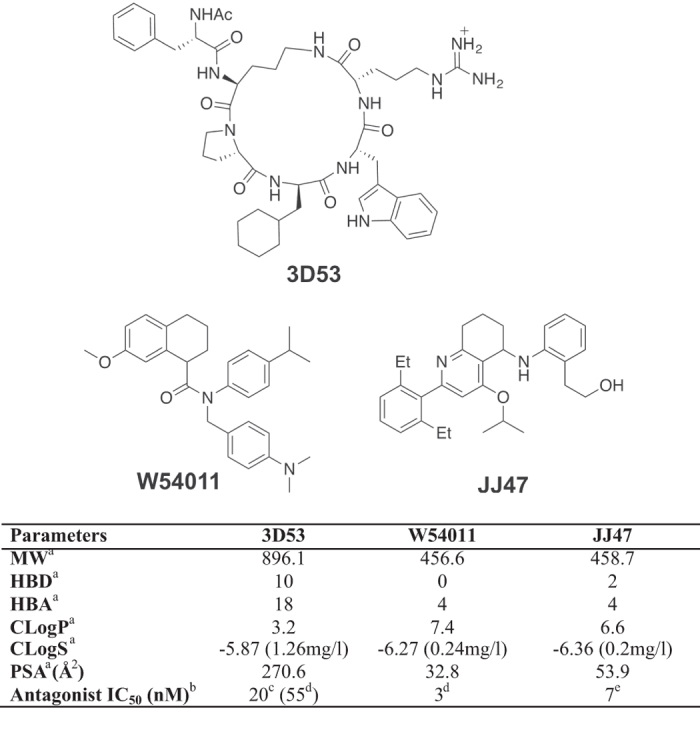
Comparative properties and *in vitro* potencies of C5aR antagonists. Top: Chemical structures for antagonists 3D53, W54011 and JJ47. Bottom: Properties and antagonist potencies of the three compounds. ^a^MW = molecular weight, HBD = hydrogen bond donors, HBA = hydrogen bond acceptors, ClogP = calculated octanol-water partition coefficient, CLogS = calculated aqueous solubility, PSA = Polar surface area. ^b^Inhibition of Ca^2+^ release in different cells, under different conditions and against different concentrations of C5a. ^c^Versus 100 nM rhC5a on neutrophils^12^. ^d^Versus 0.1nM rhC5a on neutrophils^14^. ^e^Versus 1.5 nM rhC5a on U937 cells^15^.

Complement protein C5a plays important amplification roles in recruiting immune cells to sites of infection and in inducing release of inflammatory cytokines and other mediators. When C5a formation continues unabated or its receptor C5aR is not effectively regulated, it can lead to a diverse range of multiple pathological conditions, including anaphylactic shock, sepsis and arthritis^19^. An orally bioavailable small molecule antagonist of the by-product C5aR could avoid this complication and have therapeutic benefit for treating chronic inflammatory diseases. Three compounds 3D53, W54011 and JJ47 (Fig. 1) have been reported to be non-covalent antagonists of C5a interacting with C5aR at low nanomolar concentrations, albeit measured under different conditions on different cells. A detailed comparative study here has identified a major impediment in the development of small molecule C5aR antagonists appropriate for clinical trials and the importance of carefully considering often overlooked biological principles, in this case receptor residence time of a drug, in favour of more fashionable and better known rules for drug development.

**Figure 2:**
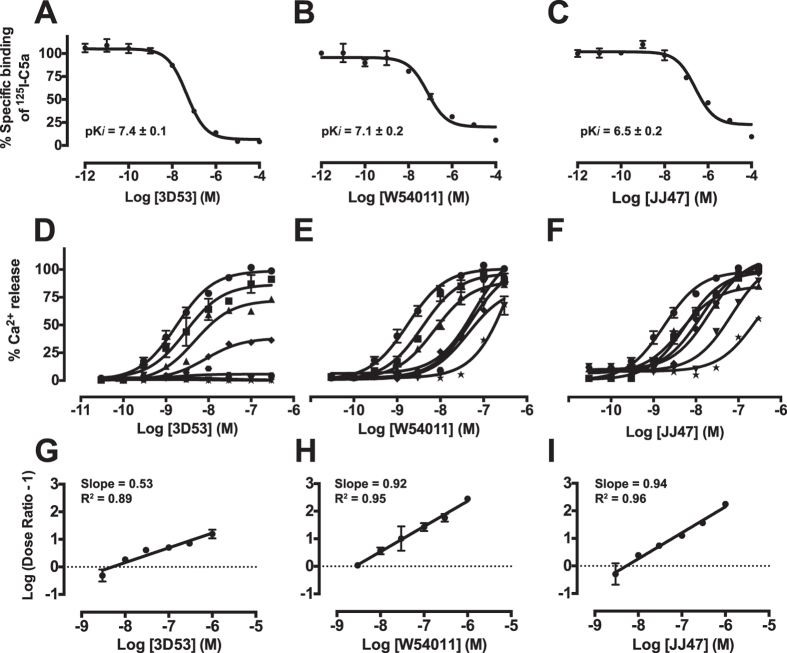
Different mechanisms of C5aR antagonism by 3D53, W54011 and JJ47 against human C5a on human monocyte-derived macrophages. Top row: Binding affinities of (**A**) 3D53, (**B**) W54011 and (**C**) JJ47 measured by displacement of [^125^I]-C5a (25 pM) from HMDM. Middle row: Concentration dependent responses to C5a following treatment with antagonist (**D**) 3D53, (**E**) W54011 and (**F**) JJ47 at various concentrations (0 nM, ●; 3 nM, ■; 10 nM, ▲; 30 nM, ◆; 100 nM, 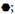 300 nM, ▼; 1000 nM, ★) with C5a (300 nM) as 100% response on human macrophages in a calcium release assay. Bottom row: Schild plots for antagonists (**G**) 3D53, (**H**) W54011 and (**I**) JJ47 against rhC5a. Calculated pA_2_ values are 8.3 (3D53), 8.6 (W54011) and 8.3 (JJ47). Error bars are means ± SEM of three independent experiments (n = 3).

**Figure 3 f3:**
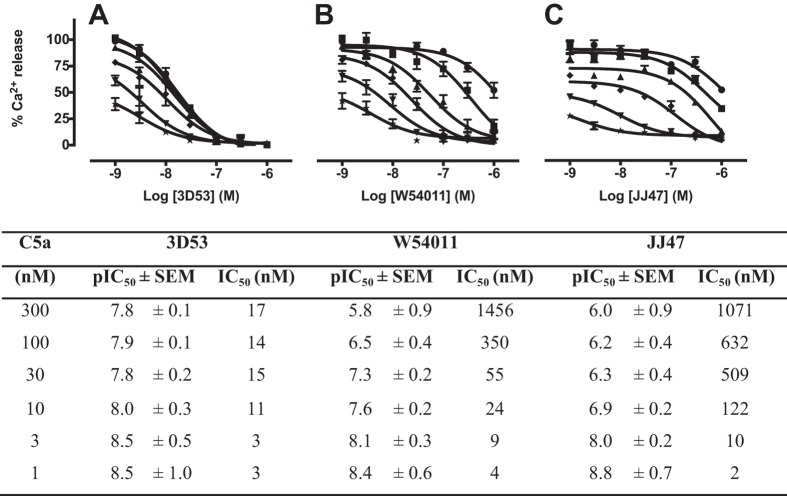
Dependence of antagonist potencies (IC_50_) on C5a concentration in human monocyte-derived macrophages. Inhibitory responses of antagonist (**A**) 3D53, (**B**) W54011 and (**C**) JJ47 against various concentrations of C5a (300 nM, ●; 100 nM, ■; 30 nM, ▲; 10 nM, ◆; 3 nM, ▼; 1 nM, ★) relative to C5a (300 nM) inducing calcium release in HMDM (100%). Calculated pIC_50_ ± SEM and IC_50_ values for the three C5aR antagonists are summarized in the table versus concentration of C5a. Error bars are means ± SEM of three independent experiments (n = 3).

First, a key point often overlooked is that IC_50_ is a concentration- and mechanism-dependent term. IC_50_ values for antagonists can only be compared against the same concentration of agonist on the same cell type under identical experimental conditions. Differences shown here for the three antagonists have very important implications (Figs 2 and 3). Cyclic peptide 3D53 was an insurmountable non-covalent binding antagonist of C5aR on HMDM with an IC_50_ almost independent of C5a concentration between 0.1 nM to 300 nM, whereas W54011 and JJ47 were competitive and surmountable non-covalent binding antagonists of C5aR with IC_50_ values that varied by three orders of magnitude against 0.1 nM to 300 nM C5a (Figs 2, [Fig f3] and 3). Serum levels of C5a can be as high as 10–100 nM in patients with sepsis^19^ and the local concentration of C5a is also expected to be high at sites of inflammation, although this remains to be documented. The results of this study question whether competitive or surmountable antagonists can even be effective therapeutic agents in the clinic for treating C5a-mediated diseases.

**Figure 4:**
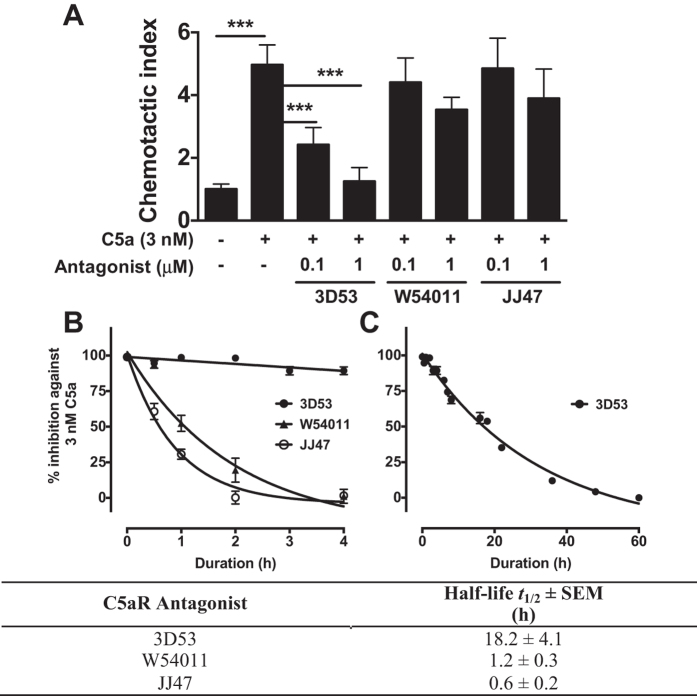
3D53 is more effective than W54011 and JJ47 in inhibiting C5a-induced migration or calcium release for human HMDM due to longer residence time on C5aR. (**A**) Migration of HMDM through 5 μm Transwell inserts with or without 3 nM C5a for 16 h. For antagonism of C5aR, cells were treated with 3D53 (0.1 or 1 μM), W54011 (0.1 or 1 μM) or JJ47 (0.1 or 1 μM) for 1 h. Unbound antagonists were washed away prior to stimulation with 3 nM C5a. Chemotactic index is expressed as fold change against control. (**B**) C5a induced Ca^2+^ release from HMDM, pre-treated with 1 μM C5aR antagonist (●) 3D53, (▲) W54011 and (○) JJ47 for 1 h. Excess unbound antagonists were removed by washing. Antagonist residence time on C5aR was determined by subjecting treated cells to 3 nM C5a during the stated duration post antagonist-treatment up to 4 h and (**C**) up to 60 h for 3D53 in a calcium release assay. Calculated half-life for 3D53, W54011 and JJ47 are 18.2 h, 1.2 h and 0.6 h respectively. Error bars are means ± SEM of three independent experiments (n = 3). ***p < 0.001 by student *t*-test.

**Figure 5:**
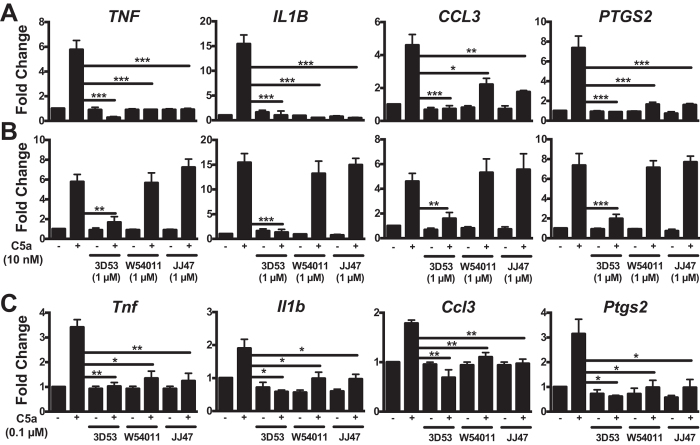
Antagonism of C5a-induced gene expression in human and rat macrophages by 3D53, W54011 and JJ47. **(A,B)** HMDM were stimulated with 10 nM C5a and lysed after 30 min. For antagonism of C5aR, HMDM were pretreated with 1 μM 3D53, W54011 or JJ47 for 1 h. Excess unbound antagonists were removed by washing and subjected to 10 nM C5a stimulation after (**A**) 1 h or (**B**) 16 h post-treatment with antagonist. (**C**) Rat macrophages were stimulated with 0.1 μM C5a and cells are lysed after 30 min. For antagonism of C5aR, cells were pretreated with 1 μM 3D53, W54011 or JJ47 for 1 h. Excess unbound antagonists were removed by washing and subjected to C5a treatment after 1 h post-treatment with antagonist. Gene expression was measured by real-time PCR, normalized against *18S* and converted to fold change relative to control. Error bars are means ± SEM of three independent experiments (n = 3). *p < 0.05, **p < 0.01 and ***p < 0.001 by student t-test.

**Figure 6:**
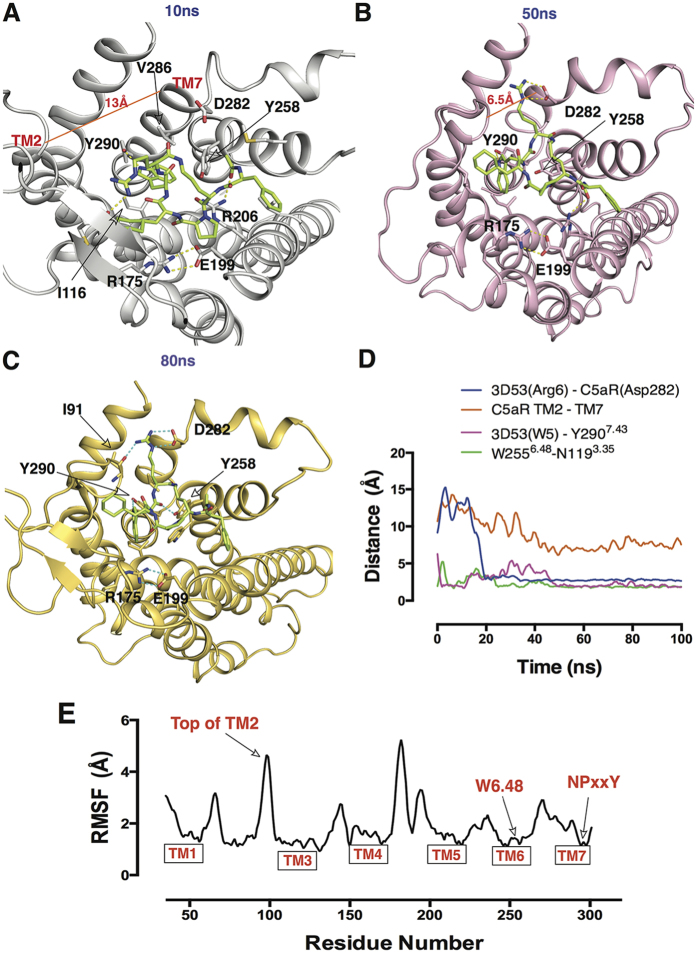
Representation of C5aR-3D53 interactions at multiple time points in MD simulations. C5aR-3D53 complex at (**A**) 10 ns: Arg6 of 3D53 faced towards ECL2 and made a short-lived contact with the Cys188 backbone carbonyl oxygen. (**B**) 50 ns: Arg6 underwent a large shift involving re-location and formation of an electrostatic interaction with Asp282, accompanied by movement of the top region of TM2 to shorten the distance to TM7. (**C**) 80 ns: similar protein-ligand interactions as at 50 ns, reflecting stable and long-lasting interactions between 3D53 and C5aR. Hydrogen bonds are depicted as dotted lines. (**D)** Distance measurements between stated residues and TM helices throughout MD simulations. (**E**) Root mean square fluctuation (RMSF) of C5aR residue sidechains throughout MD simulations.

A second key point concerns antagonist residence time on its target receptor. Washout experiments used here (Figs 2, 3, 4, 5) showed that the more drug-like compounds W54011 and JJ47 exerted their effects on macrophages for much shorter durations than 3D53 (ca. *t*_1/2_ ≤ 2 h vs 20 h), irrespective of the functional readout measured (Ca^2+^ release, inflammatory gene expression, chemotaxis). The striking differences in duration of action, being an order of magnitude longer for 3D53 than for W54011 and JJ47, reflects a much longer residence time on the receptor C5aR for 3D53. This increased residence time was linked via molecular dynamics simulations of the antagonist-receptor complex to a ligand-induced conformational change that results in the antagonist being uniquely trapped, relative to two other competitive antagonists, in the receptor between TM helices (Fig. 6). An insurmountable antagonist with long residence time such as 3D53 could be advantageous in systems with rapid and transient signalling^31^,^32^. Generation of C5a is localized at the membrane during inflammation and can be profoundly high for brief but repeated periods. A rapidly dissociating antagonist (W54011 or JJ47) would be less efficacious than a more slowly dissociating antagonist (3D53) that remains bound to C5aR even in the presence of high C5a concentrations. Thus 3D53 would require only once daily administration, whereas W54011 and JJ47 would be required multiple times a day and in much larger doses. The important advantages conferred by longer residence time are not widely appreciated and often overlooked in drug design and development. The importance of receptor residence time of a drug has begun to be better appreciated for kinases, with most marketed kinase inhibitors until recently having short residence times^33^. This has in part led to development of covalent kinase inhibitors^34^, and there has been a resurgence of interest in covalent drugs more generally^35^.

**Figure 7:**
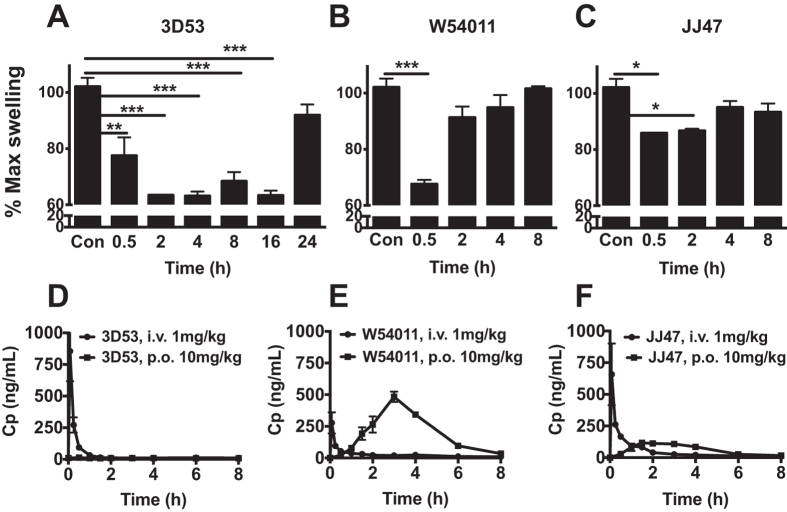
3D53, W54011 and JJ47 are orally active anti-inflammatory compounds that inhibit C5a agonist-induced paw oedema in male Wistar rats. Paw oedema was induced in male Wistar rats by intraplantar administration of C5aR-PA (350 μg per paw in 100 μL of saline control). Paw swelling (% area change from baseline) was recorded at indicated timepoints. To determine comparative *in vivo* residence times, (**A**) 3D53 (5 mg/kg, p.o., n = 3 per group), (**B**) W54011 (30 mg/kg, p.o., n = 2 per group) and (**C**) JJ47 (30 mg/kg, p.o., n = 2 per group) were given orally 1 h prior to injection of C5aR-PA at specific time-points post-antagonism. Oral bioavailabilities of (**D**) 3D53 (F ~ 2%), (**E**) W54011 (F ~ 74%) and (**F**) JJ47 (F ~ 12%) were measured using LCMS to quantify plasma concentrations. C5aR antagonist was administering as an intravenous dose (1 mg/kg via an implanted jugular vein catheter in 50:50 DMSO/saline) versus an oral dose (10 mg/kg in olive oil, via gavage). Plasma was collected at various time points and analyzed. Error bars are means ± SEM normalized to maximal swelling of saline control. *p < 0.05, **p < 0.01 and ***p < 0.001 by student *t*-test.

A third important point concerns a very misleading pharmacokinetic measurement, F% or oral bioavailability. Since drugs that are administered orally or via other routes have to survive degradative and metabolizing enzymes, cross membrane barriers, and be cleared only slowly from the circulation in order to exert systemic drug action, the degree of oral bioavailability is usually considered to be of paramount importance in drug development. Indeed rule-of-five compliance^1^,^2^,^3^ in medicinal chemistry has completely changed the direction of drug discoverers and the chemical space in which they operate over the past two decades. F% is the fraction of orally administered drug in systemic circulation unchanged relative to its intravenously administered concentration defined as 100%. The higher F%, the lower the dose needed for therapeutic effect and this in turn reduces risks of side effects or off-target effects and toxicity. Drugs with low F% on the other hand can result in low oral efficacy, requiring higher doses, which in conjunction with high patient variability can lead to unpredictable drug responses. It is almost a paradigm in clinical settings that patient health must be correlated with circulating drug levels. However, this notion is fundamentally flawed if a drug has a fast on-rate, is cleared quickly from blood, and has a slow off-rate due to being bound to its receptor for extended durations. For example, 3D53 has a very low F% and high clearance rate versus W54011 and JJ47 (Fig. 7). Despite its inferior oral bioavailability, supposedly reflecting a much smaller amount of drug exerting its effect on its receptor, 3D53 is clearly the superior antagonist *in vivo* with a 10-fold longer duration of action *in vivo* and much greater potency in the presence of escalating concentrations of C5a (Fig. 7).

This study has demonstrated that receptor residence time of a non-covalent binding antagonist measured *in vitro* can relate to duration of action and degree of efficacy *in vivo*. Drug discovery has traditionally focused on optimizing drug affinity for a receptor, functional potency, receptor selectivity and drug-like chemical components in order to maximize pharmacokinetics and oral bioavailability. However, these properties do not necessarily maximize *in vivo* efficacy, as highlighted herein. Fast plasma clearance combined with low F% is commonly interpreted as resulting in low efficacy, a conclusion that can be incorrect as shown here. The affinity of a ligand for its receptor does not, *per se*, define the effectiveness and duration of biological action. Rather, it is the lifetime of the binary receptor-ligand complex that in part dictates the effect in the cellular and organismal context. If the drug is circulating in blood, unbound to its receptor, it is not exerting its effect and this needs to be considered more carefully during drug optimization. Antagonist action is certainly related to binding to its target, but the longer the receptor-antagonist complex is maintained intact, the longer the antagonist effect. This fundamental property of ligand residence time on the receptor is not a new concept^36^,^37^, but perhaps is under-appreciated. The recent resurgence of interest in covalent binding drugs^35^ has not yet been matched by efforts to create non-covalent drugs with longer residence times.

The value of receptor residence time has been dramatically highlighted here for an extreme case of a molecule that violates physicochemical parameters traditionally used to characterize drug-likeness. Even though 3D53 has low oral bioavailability (Fig. 7D) it has a substantial oral activity and outperforms much more drug-like and more orally bioavailable antagonists of comparable *in vitro* potency when compared *in vivo* as an orally administered anti-inflammatory compound. As drug discovery researchers, developers and translators work through the tremendous challenges in taking effective and safer drugs to the market, perhaps one of the larger problems is a less realized one, that we may have lost sight of the importance of fundamental biological principles. Time is of the essence in drug development, and also for drug efficacy where residence time of a drug on its specific receptor can be critical in determining the duration and effectiveness of drug action.

## Methods

### Reagents

Recombinant human M-CSF (PeproTech, USA), recombinant human C5a (Sino Biological Inc, China), cell culture reagents (Invitrogen, Australia) and analytical grade chemical reagents (Sigma-Aldrich, Australia) were from commercial sources. C5aR antagonists (W54011, JJ47, 3D53) and C5aR peptide agonist (C5aR-PA) Ac-Phe-Lys-Pro-D-Cha-Cha-D-Arg-OH (modified with N-acetyl cap) were synthesized and characterized (analytical HPLC, mass and nuclear magnetic resonance spectroscopy) in-house as described^13^,^14^,^15^,^38^.

### Isolating human monocyte-derived macrophages

CD14^+^ human monocytes were isolated as described^5^. Briefly, monocytes were isolated from buffy coats of anonymous donors (Australian Red Cross Blood Service, Australia) by Ficoll-Paque Plus (GE Healthcare). Contaminating erythrocytes were removed by repeated washing with ice-cold sterile water. CD14^+^ MACS^®^ microbeads (Miltenyi Biotec) were used to isolate CD14^+^ monocytes, and cells were cultured in IMDM supplemented with 10% FBS, 10 U/mL penicillin/streptomycin and 2 mM GlutaMAX with 10 ng/mL M-CSF for 6 days to mature them to human monocyte-derived macrophages (HMDM).

### Binding affinity assay

A scintillation proximity assay was used to measure the affinity of C5aR antagonists as described^39^. HMDM were incubated for 1 h with the specified concentration of C5aR antagonist, polyvinyltoluene scintillation beads were coated with wheat germ agglutinin (PerkinElmer) and 25 pM of [^125^I]-C5a. Radioligand binding was then analyzed using a Microbeta scintillation counter (PerkinElmer).

### Intracellular calcium release assay

Intracellular calcium release was induced in HMDM as described^40^ and measured using a FLIPR instrument (Molecular Devices). HMDM were seeded in multiple 96-well plates for each time point and allowed to adhere overnight. 3D53, W54011 and JJ47 were prepared in DMSO as 10 mM stock and dilutions were performed in HBSS. To investigate antagonist residence time on C5aR, HMDM were first pre-treated with each C5aR antagonist (1 μM) for 1 h at 37 °C. After 1 h, excess unbound antagonists were removed by washing with HBSS and the antagonist-treated HMDM were then challenged by stimulating with rhC5a (3 nM) after different incubation times (0, 0.5, 1, 2, 4, 6, 8, 16, 22, 36, 48, 60 h) to compare the residence time of antagonist.

### *In vitro* migration assay

HMDM were serum-deprived overnight prior treatment with C5aR antagonist (0.1 or 1 μM) for 1 h at 37 °C. Excess unbound antagonists were washed with serum-free medium and HMDM (0.1 × 10^6^ cells/mL) then seeded into 5 μm Transwell inserts (Corning) creating a modified Boyden chamber. To initiate cell migration, serum-free medium with rhC5a (3 nM) was added to the lower chamber and incubated for 16 h at 37 °C. Migrated cells on the lower side of the membrane were stained with DAPI and counted. The chemotactic index was expressed as fold change versus control.

### Gene expression analysis

RNA was extracted from cells using ISOLATE II RNA Kit (Bioline) following manufacturer’s instructions. Real-time PCR was performed using 7900HT real-time with SYBR^®^ Green PCR master mix (Applied Biosystems) and primers. Relative gene expression was normalized against *18S* rRNA expression and expressed as fold change against control samples. Primer sequence is shown in Supplementary Table 2.

### Homology modelling and docking of C5aR

GPCRDB^44^ (http://www.gpcr.org/7tm/) was searched for the best template structure for building a homology model of C5aR. At the time of the model building, CXCR4 (PDB code: 3ODU) was the top template (Sequence Identity = 32%; Sequence Similarity = 53%) (Supplementary Fig. 6). A Ramachandran structural plot suggested that the homology model of the TM region of C5aR based on CXCR4 was both stereochemically and energetically reasonable (Supplementary Fig. 7). The antagonist bound structure of CXCR4 serves a reasonable starting template. Initial sequence alignment was performed at the TM-coffee website. Further alignment was adjusted using Jalview program. Homology models were built with Modeller software with the selection of the final model gaining overall high score from both DOPE and GA341. The whole ECL2 was further refined using the loop refinement option (default setting) in Prime (version 3.1, Schrödinger, LLC, New York, NY, 2012) and the final model that incorporated the optimized ECL2 was minimized in Prime using the truncated-Newton energy minimization (OPLS_2005 force field with restrained helical backbone). The “structure assessment” module available at Swiss-Model portal (http://swissmodel.expasy.org/) was used for local model quality estimation (QMean Score) global model quality estimation (DFIRE energy) and stereochemistry check (Procheck)^45^,^46^. The NMR solution structure^13^ of 3D53 was docked into the C5aR homology model using GOLD software with no constraints in order to find all possible poses that might agree with previous modeling and experimental results^22^. The top docked pose (Supplementary Fig. 8) reflected a similar docking mode to previous modeling for C5aR and 3D53, where Arg6 interacted with Asp282 at the top of TM7 near to ECL3.

### Molecular dynamics simulations of C5aR-3D53 complex

Using the top docking pose, 100 ns MD simulations for C5aR-3D53 complex were investigated. The whole system consisting of the docked 3D53 in C5aR was first prepared with standard protocol in “Protein Preparation Wizard” within Maestro, version 9.5 (Schrödinger, LLC, New York, NY, 2013). Hydrogen atoms were added, and hydrogen bond assignment, tautomer, and protonation of amino acids at pH 7.4, were optimized. The prepared structure was then embedded in 1-palmitoyl-2-oleoyl-sn-glycero-3-phosphocholine (POPC) membrane via membrane set up panel in Desmond Molecular Dynamics System v3.5 (D. E. Shaw Research, New York, NY, 2013). TIP3P water was used for system solvation. Neutralization of the system was implemented by addition of Cl- ions at physiological concentration of 0.15M. After solvating the whole system, a membrane protein relaxation protocol was generated and used for further simulation with Berendsen coupling. The all-atom optimized potential for liquid simulations (OPLS-2005) force field implemented as the default force field in Desmond was adopted for all molecules in the system. Prior to the MD production, two steps of minimization were applied. First step included minimization with restraints on solute heavy atoms with a force constant of 50 kcal/mol/Å^2^. Second step was the minimization without any restraints. The minimized system was further relaxed before the actual simulation. Six steps were included: 1) heating up system to 300 K with NVT ensemble with Berendsen coupling for 60 ps. 2) 200 ps equilibration NPT restrained on heavy atoms. 3) NPT equilibration of solvent and lipids for 100 ps. 4). NPT with protein heavy atoms for 600 ps. 5) NPT equilibration with protein c alpha atoms at 2 kcal/mol. 6) NPT with no restraints for 100 ps. Finally, MD run stage was performed with NPT ensemble at 300 K temperature and 1.01325 bar for pressure. Berendsen coupling for both thermostat and barostat was adopted with an integration of 2 fs. Coulombic interactions were calculated using a cutoff radius 9 Å. Long range electrostatical interactions were calculated with Particle Mesh Ewald (PME) method.

#### Animals

Male Wistar rats (6–8 wk, 250 ± 50 g) were bred at the Animal Resources Centre (Canning Vale, Australia) and housed post-shipment at the Australian Institute for Bioengineering and Nanotechnology (University of Queensland, Brisbane). Animals were maintained in a 12 h light-dark cycle with food and water provided. Male Wistar rats were given 3D53 (5 mg/kg, *n* = 3/group) or W54011 or JJ47 (30 mg/kg, *n* = 2/group) (p.o. *via* gavage in 500 μL olive oil) prior to administering C5aR-PA. Control animals received only olive oil (500 μL p.o.). After specific time-points, C5aR-PA was administered into the right hind paw pad (350 μg in 100 μL saline, i.pl). The left hind paw acted as a control, receiving saline only (100 μL). Rear paw thickness and width were measured at 1 h (and periodically over 4 h) post-injection using digital callipers (World Precision Instruments, Sarasota, FL, USA) and swelling was expressed as area (mm^2^; thickness × width) and plotted as percentage change from baseline of each individual paw. The maximal swelling induced by 350 μg C5aR-PA maximized after 1 h before subsiding after 4 h following injection. Higher doses of C5aR-PA (1 mg and 2 mg) did not further increase the magnitude of paw swelling, so 350 μg was used as optimal dose of C5aR-PA. Oral bioavailability was determined in male Wistar rats after administering either an intravenous dose (1 mg/kg via an implanted jugular vein catheter in 50:50 DMSO/saline, *n* = 4; 3D53, *n* = 4; W54011 and *n* = 2; JJ47) or an oral dose (10 mg/kg in olive oil, via gavage). Plasma was collected at various time points (0, 5, 15, 30 min, 1, 2, 3, 4, 6 and 8 h) from the jugular vein catheter and analyzed for antagonist concentrations using LCMS as described^41^. The animal ethics committee of The University of Queensland approved all experiments according to NHMRC guidelines for the care and use of laboratory animals and in accordance with ARRIVE guidelines for reporting experiments involving animals^42^,^43^. A total of N animals were used in each experiment as described.

### Statistical analysis

Data were plotted and analyzed using GraphPad Prism (5.0 d) for Mac OS X (GraphPad Software). Statistically significant differences were assessed using student’s t-test for paired comparison. All values of independent parameters were mean ± SEM of at least three independent experiments. *p < 0.05, **p < 0.01 and ***p < 0.001.

## Additional Information

**How to cite this article**: Seow, V. *et al.* Receptor residence time trumps drug-likeness and oral bioavailability in determining efficacy of complement C5a antagonists. *Sci. Rep.* **6**, 24575; doi: 10.1038/srep24575 (2016).

## Supplementary Material

Supplementary Information
