# Supplementary material for: Receptor residence time trumps drug-likeness and oral bioavailability in determining efficacy of complement C5a antagonists
Source: Sci Rep. 2016 Apr 20;6:24575. doi: 10.1038/srep24575 (PMC4837355; doi:10.1038/srep24575)
Supplement: Supplementary Information [file srep24575-s1.pdf]

Supplementary Information

Receptor residence time trumps drug-likeness and oral bioavailability in determining efficacy of complement C5a antagonists

Vernon Seow[1a†], Junxian Lim[1a,2,3†], Adam J. Cotterell[1a], Mei-Kwan Yau[1a,2,3], Weijun Xu[1a,2,3], Rink-Jan Lohman[1a,2,3], W. Mei Kok[1a,2,3], Martin J. Stoermer[1a], Matthew J. Sweet[1b,2,3], Robert C. Reid[1a,2,3], Jacky Y. Suen[1a,2,3], David P. Fairlie[1a,2,3]*

[1a]Division of Chemistry and Structural Biology or [1b]Division of Cell Biology and Molecular Medicine, Institute for Molecular Bioscience, The University of Queensland, Brisbane, QLD 4072, Australia.

[2]Centre for Inflammation and Disease Research, Institute for Molecular Bioscience, The University of Queensland, Brisbane, QLD 4072, Australia.

[3]ARC Centre of Excellence in Advanced Molecular Imaging, Institute for Molecular Bioscience, The University of Queensland, Brisbane, QLD 4072, Australia.

[†]These authors contributed equally.

***Correspondence to:** Professor David P. Fairlie (Tel: +61-7334 62989, Fax: +61-7334 62990, Email: d.fairlie@imb.uq.edu.au)

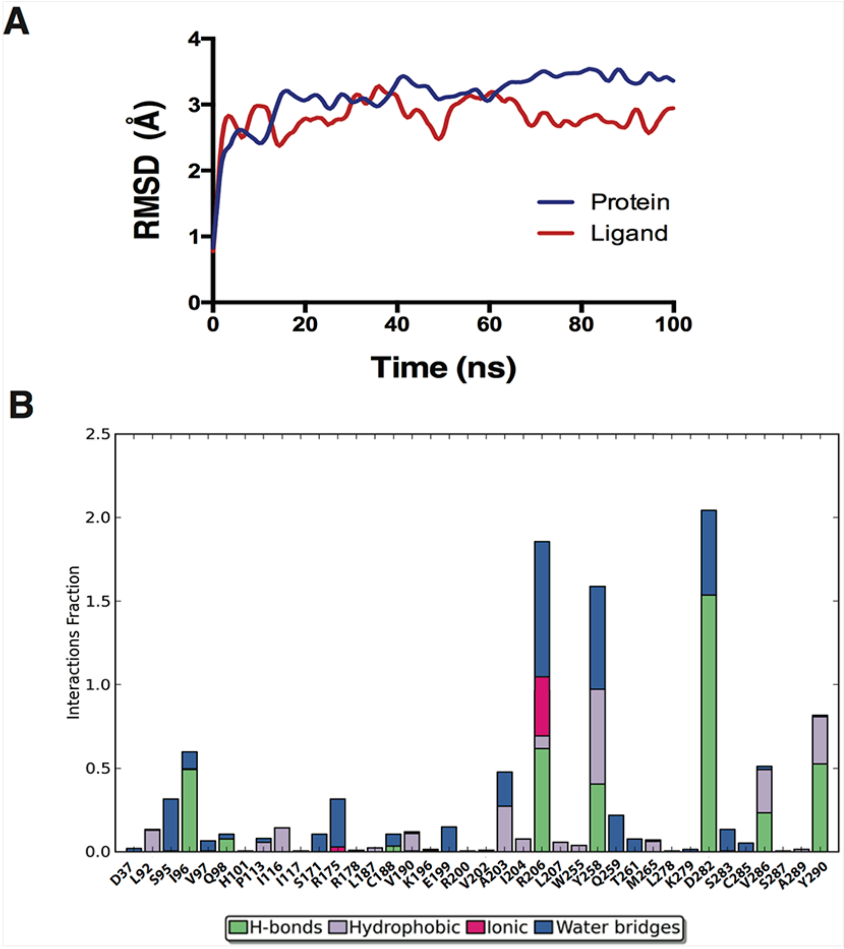

Supplementary Figure 1. Statistics from MD simulations. (**A**) C5aR-3D53 root mean square deviation (RMSD) over 100 ns MD simulations. (**B**) C5aR-3D53 interaction intensities plot.

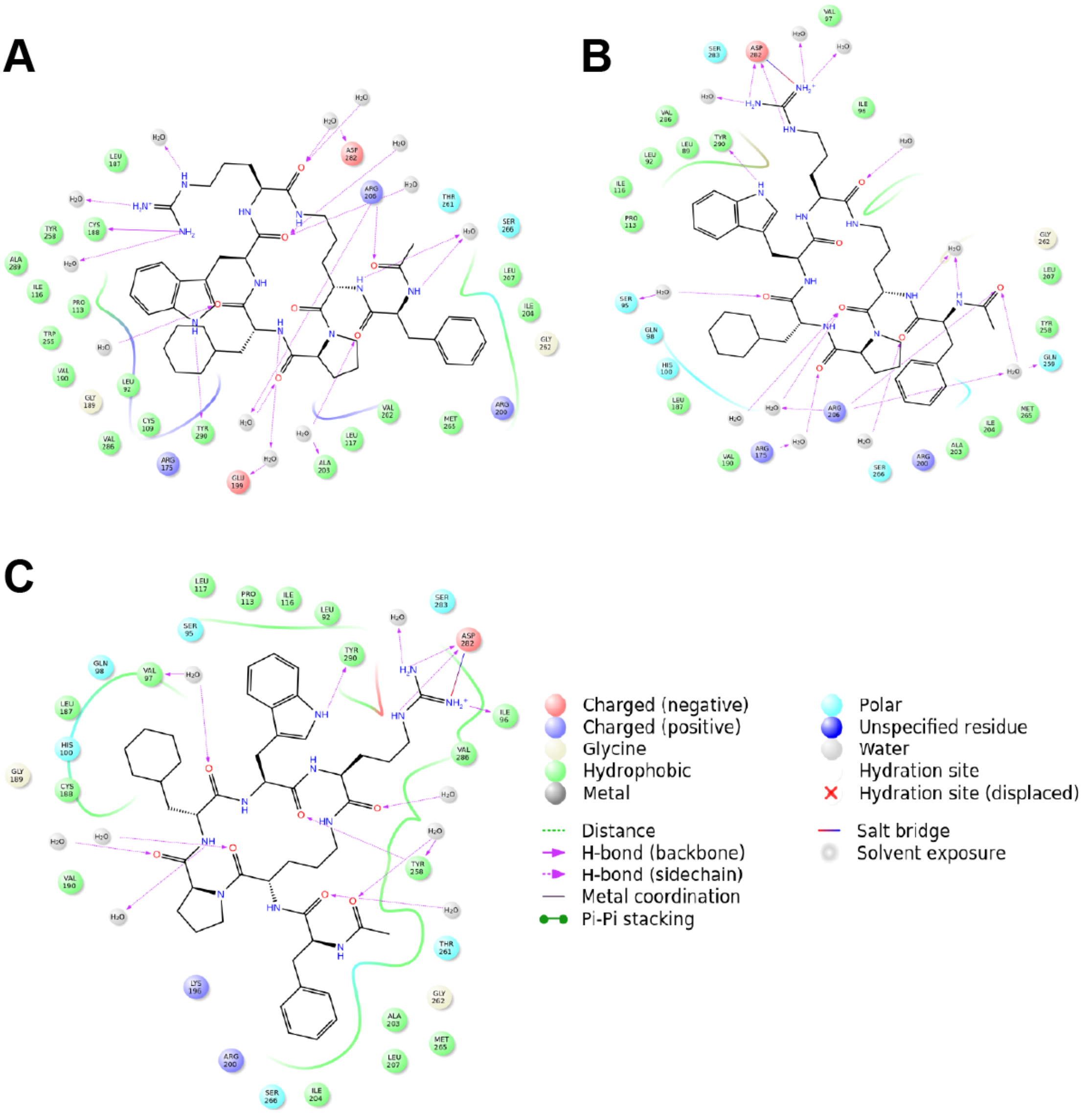

Supplementary Figure 2. Two-dimensional representations of 3D53-C5aR interactions at multiple time points in MD stimulations. (**A**) C5aR-3D53 complex at 10 ns, (**B**) 50 ns (**C**) and 80 ns.

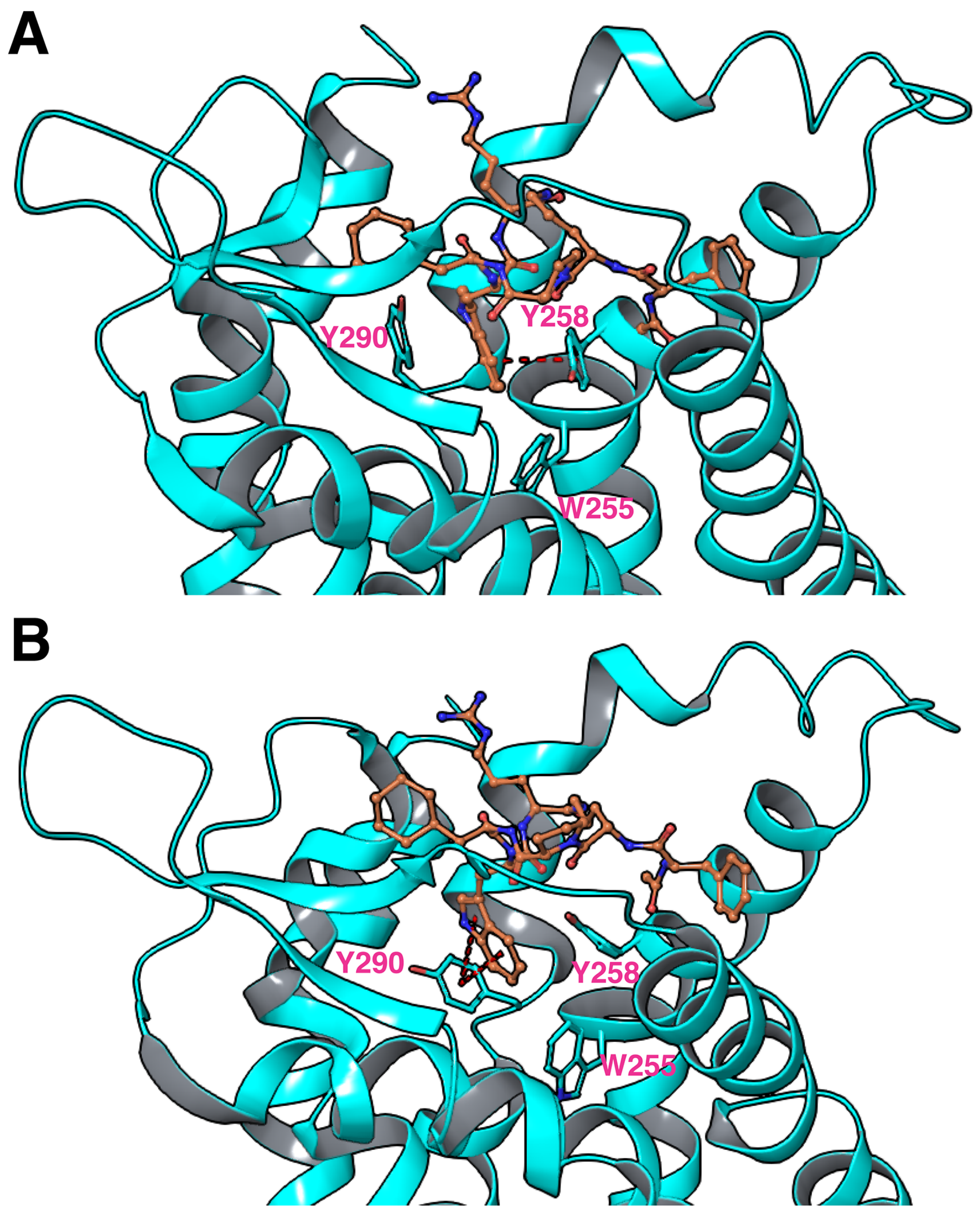

Supplementary Figure 3. **C5aR-3D53 interactions at 25 ns and 45 ns**. (**A**) 25 ns, Trp5 pi-stacked between TM6 (Tyr258) and TM7 (Tyr290). (**B**) 45 ns, Trp5 moved out to form H-bond with Y290, with an intermediate stage of forming a T-shaped edge-to-face pi-stacking interaction with Y290.

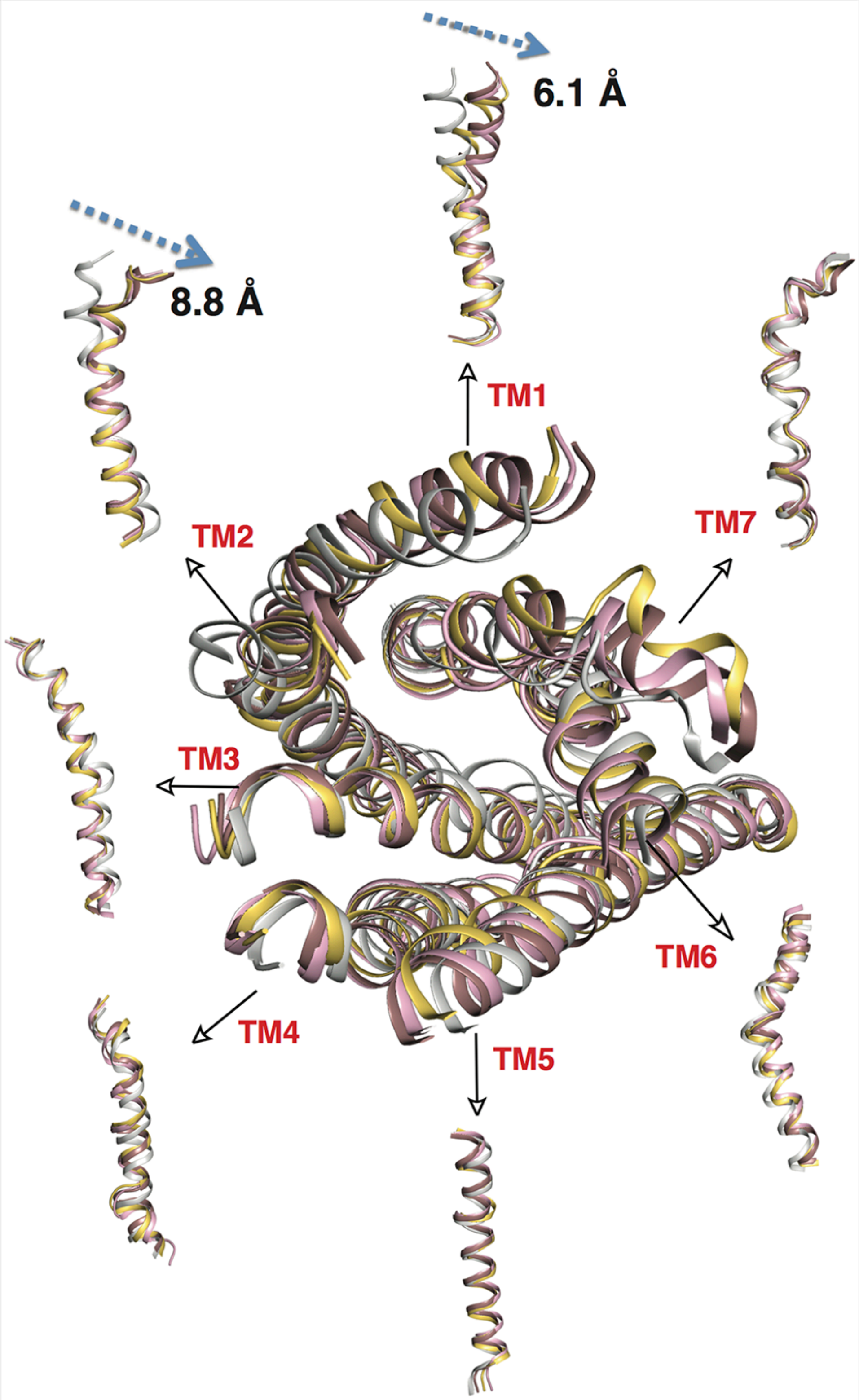

Supplementary Figure 4. Comparison of relative conformational change in C5aR transmembrane. Four different time points were sampled during 3D53-C5aR MD simulations (3D53 ligand was removed for clarity), white: 10 ns; pink: 50 ns; gold: 80 ns and brown: 100 ns. Transmembrane (TM) 1 and 2 helices were observed to undergo significant conformational changes in the MD simulations of the 3D53-C5aR complex, moving 8.8 Å and 6.1 Å respectively.

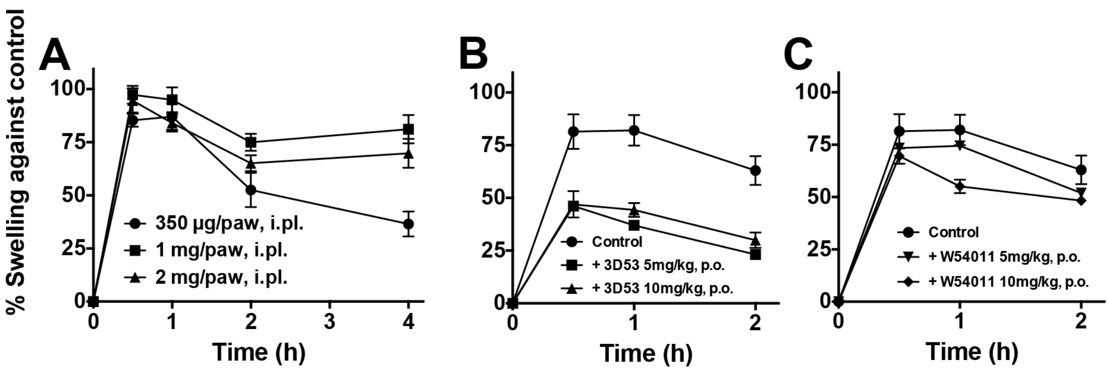

Supplementary Figure 5. Optimization for C5aR-PA-induced paw oedema in male Wistar rats. (**A**) Intraplantar (i.pl.) administration of C5aR-PA (0.35-2 mg per paw in 100 µL of saline control. Paw swelling was recorded over 4 h duration and expressed as percentage area change from baseline. The maximal swelling induced by 350 µg C5aR-PA maximized after 1 h before subsiding after 4 h following injection. Higher doses of C5aR-PA (1 mg and 2 mg) did not further increase the magnitude of paw swelling, so 350 µg was considered an optimal dose of C5aR-PA for injection per paw. (**B**) 3D53 and (**C**) W54011 (5 and 10 mg/kg, p.o. in 500 µL olive oil per rat) given orally 1 h prior 350 µg C5aR-PA injection. Error bars are means ± SEM (n=2 per group).

```
CLUSTAL W (1.83) multiple sequence alignment

sp|P61073|CXCR4_HUMAN  MEGISIYTSDNYTEEMGSGDYDSMKEPCFREENANFNKIFLPTIYSIIFLTGIVGNGLVILVMGYQKKLRSMTDKYRLHL 80
sp|P21730|C5AR1_HUMAN  MDSFNYTTP-DYGHYDDKDTLDLNTPVDKTSNTLRVPDILALVIFAVVFLVGVLGNALVVWVTAFEAK-RTINAIWFLNL 78
                       *:.:.  *. :* .  ...  *  .     .:. .. .*:  .*::::**.*::**.**: * .:: * *::.  : *:*

sp|P61073|CXCR4_HUMAN  SVADLLFVITLPFWAVD--AVANWYFGNFLCKAVHVIYTVNLYSSVLILAFISLDRYLAIVHATNSQRPRKLLAEKVVYV 158
sp|P21730|C5AR1_HUMAN  AVADFLSCLALPILFTSIVQHHHWPFGGAACSILPSLILLNMYASILLLATISADRFLLVFKPIWCQNFRGAGLAWIACA 158
                       :***:*  ::**:  ..     :* **.  *. :  :  :*:*:*:*:** ** **:* :.:.  .*. *      :. .

sp|P61073|CXCR4_HUMAN  GVWIPALLLTIPDFIFANVSE--ADDRYICDRFYPNDLWV-VVFQFQHIMVGLILPGIVILSCYCIIISKLSHSKGHQKR 235
sp|P21730|C5AR1_HUMAN  VAWGLALLLTIPSFLYRVVREEYFPPKVLCGVDYSHDKRRERAVAIVRLVLGFLWPLLTLTICYTFILLRTWSRRATRST 238
                        .*  *******.*::  * *     : :*.  *.:*     .. : ::::*:: * :.:  ** :*: :    :. :.

sp|P61073|CXCR4_HUMAN  KALKTTVILILAFFACWLPYYIGISIDSFILLEIIKQGCEFENTVHKWISITEALAFFHCCLNPILYAFLGAKFKTSAQH 315
sp|P21730|C5AR1_HUMAN  KTLKVVVAVVASFFIFWLPYQVT-----GIMMSFLEPSSPTFLLLKKLDSLCVSFAYINCCINPIIYVVAGQGFQGRLRK 313
                       *:**..* :: :**  **** :       *::.::: ..     ::*  *:  ::*:::**:***:*.. *  *:   ::

sp|P61073|CXCR4_HUMAN  ALTSVSRGSS--LKILSKGKRGGHSSVSTESESSSFHSS 352
sp|P21730|C5AR1_HUMAN  SLPSLLRNVLTEESVVRESKSFTRSTVDTMAQ--KTQAV 350
                       :*.*: *.    .:: :.*   :*:*.* ::  . ::
```

Supplementary Figure 6. ClustalW sequence alignment of human CXCR4 and C5aR1. CXCR4 was the top template with sequence identity of 32% and sequence similarity of 53% to C5aR. Uniprot ID codes are provided.

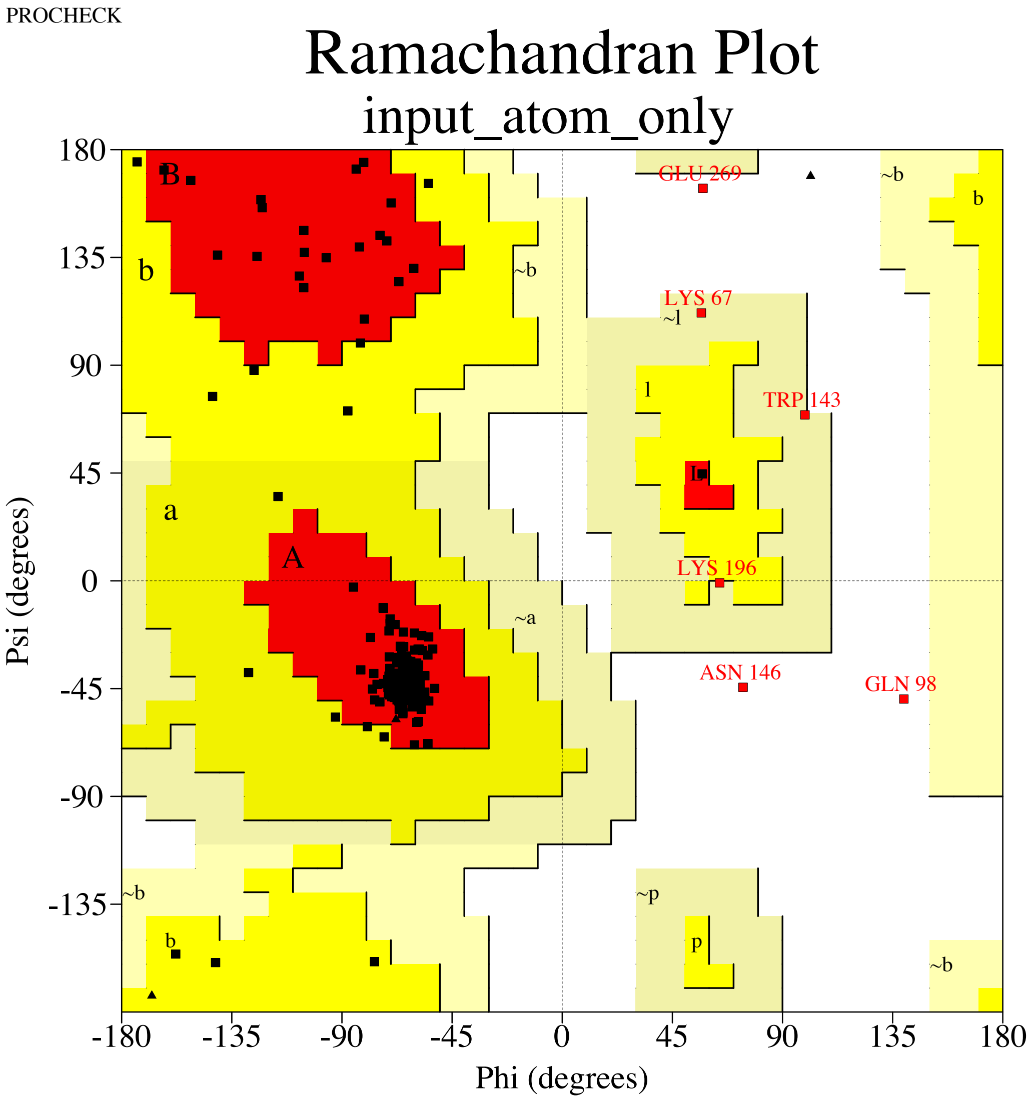

Plot statistics

Residues in most favoured regions [A,B,L]	222	91.7%
Residues in additional allowed regions [a,b,l,p]	14	5.8%
Residues in generously allowed regions [~a,~b,~l,~p]	3	1.2%
Residues in disallowed regions	3	1.2%
	----	------
Number of non-glycine and non-proline residues	242	100.0%
Number of end-residues (excl. Gly and Pro)	2	
Number of glycine residues (shown as triangles)	10	
Number of proline residues	13	

Total number of residues	267	

Supplementary Figure 7. Ramachandran Plot of stereochemical property of residues and quality assessment of the homology model of C5aR TM region. 98.7% of the residues were located in the favored and allowed φ-ψ regions, whereas only 3 residues were in disallowed region. The model of C5aR TM regions forms intact canonical seven-helical bundles. The TM backbone rmsd between the model and the template CXCR4 is 0.18 Å. The Qmean[1] score is 0.45 and DFire[2] energy is -407.7. The results were comparable to original template with a Qmean6 score of 0.48 and DFire energy of -470.

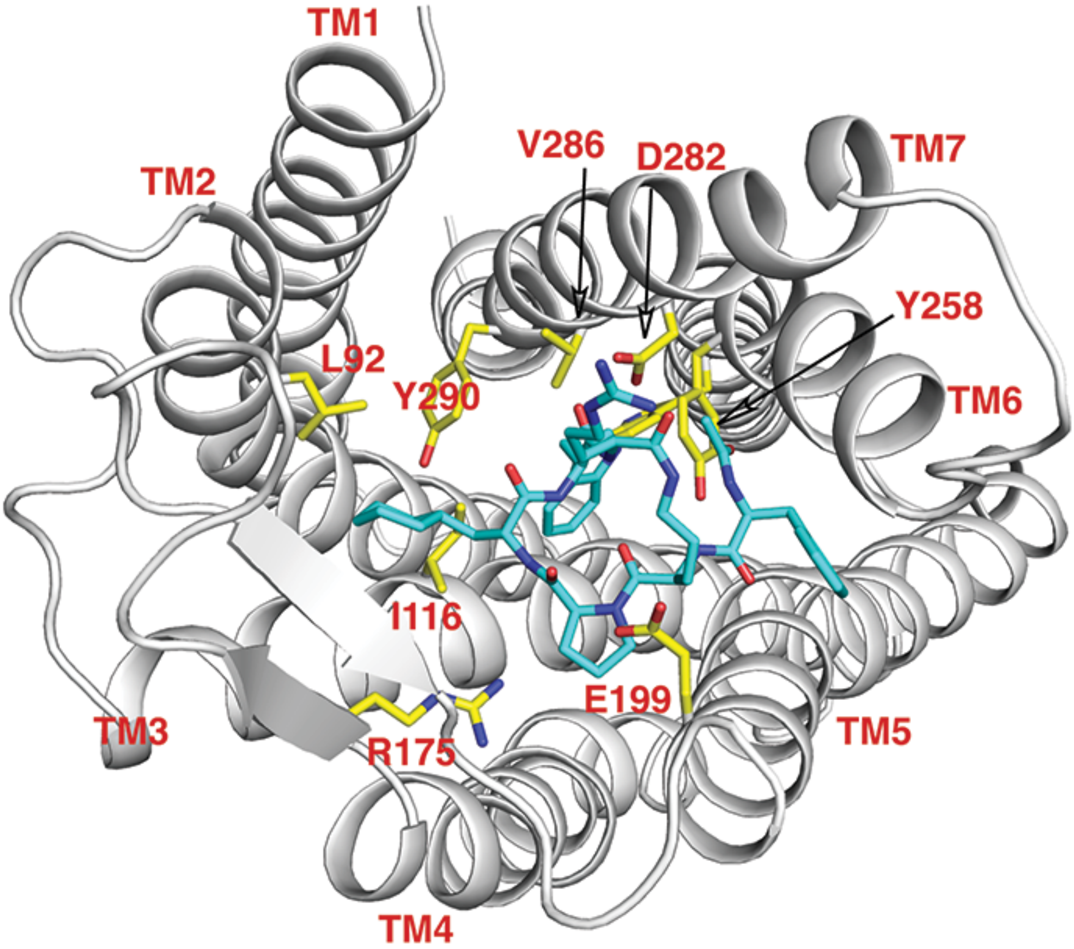

Supplementary Figure 8. Top **docked pose of 3D53 in C5aR homology model.** Docked pose of 3D53 in C5aR homology model showed similar pose to previous studies[13]. Trp5 of 3D53 is known to dictate function, switching to agonist activity if Trp was changed to a hydrophobic non-aromatic group but maintaining antagonist activity if changed to an aromatic group (Phe, Naphthalanine)[13]. Ile116 is known to be important for this activation switch blocked by Trp5[25]. On the opposite side, Val286 was also shown to contribute to the mechanism. Thus Trp5 is in the vicinity of Ile116 and Val286. Ac-Phe was predicted to locate between TM5 and TM6, there was no dominant interaction with this residue, consistent with our previous modelling studies[25]. Arg6 was not observed to form any salt-bridge to protein residues but was exposed to solvent.

Supplementary Table 1. Pharmacokinetics parameters of C5aR antagonists in rats.

	3D53[a]		W54011[a]		JJ47[a]	
	Average	SEM	Average	SEM	Average	SEM
Rat weight (g)	221.6	9.3	254	6.9	238.3	2.3
Vdss (L)	0.9	0.4	4.6	0	0.8	0.1
	4.1	1.9	18.3	3.1	3.5	0.5
AUC i.v. (0-8h) (ng.h/ml)	322.9	17.7	212.5	29.3	410.4	39.5
AUC p.o. (0-8h) (ng.h/ml)	76.6	31	1570.2	151.5	501.3	5.2
CL (ml/min/kg)	52.1	2.8	82.8	10.5	41	4
t1/2 (min)	21.6	4.4	124.4	31.3	43.2	4.8
C_{max} (ng/ml)	17.5	4.5	483.6	41.2	126.6	14.5
T_{max} (min)	50	20	180	0	135	45
F(%)	2.4	1	74.3	7.9	12.2	0.1

[a]C5aR antagonist were administered 1 mg/kg i.v. and 10 mg/kg p.o.
Vdss; volume of distribution steady
AUC; area under curve
CL; total clearance
t1/2; plasma half-life
C_{max}; maximal plasma concentration reached
T_{max}; time of maximal C_{max}
F; Fraction of intact compound absorbed to plasma (oral availability)

Supplementary Table 2. Primer sequences for target genes.

Gene	5'-3'	3'-5'
TNF	TCTGGCCCAGGCAGTCAGATC	CACTGGAGCTGCCCCTCAGC
IL1B	CCTCTTCGAGGCACAAGGCACAA	TGGCTGCTTCAGACACTTGAGCAAT
CCL3	CCCACATTCCGTCACCTGCTCAG	AGCAGCAAGTGATGCAGAGAACTG
PTGS2	TGGCAGGGTTGCTGGTGGTA	TCTGCCTGCTCTGGTCAATGGA
18S	ACCACGGGTGACGGGGAATC	CCGGGTCGGGAGTGGGTAAT
